# Changes in the microbial communities during co-composting of digestates^☆^

**DOI:** 10.1016/j.wasman.2013.12.009

**Published:** 2014-03

**Authors:** Ingrid H. Franke-Whittle, Alberto Confalonieri, Heribert Insam, Mirko Schlegelmilch, Ina Körner

**Affiliations:** a University of Innsbruck, Institute of Microbiology, Technikerstraße 25, 6020 Innsbruck, Austria; b Scuola Agraria del Parco di Monza,Viale Cavriga 3, 20900 Monza, Italy; c Hamburg University of Technology, Bioconversion and Emission Control Group, Eissendorfer Str. 42, 21073 Hamburg, Germany

**Keywords:** Composting, Anaerobic digestion, COMPOCHIP, Microarray, Nitrogen compounds

## Abstract

Anaerobic digestion is a waste treatment method which is of increasing interest worldwide. At the end of the process, a digestate remains, which can gain added value by being composted. A study was conducted in order to investigate microbial community dynamics during the composting process of a mixture of anaerobic digestate (derived from the anaerobic digestion of municipal food waste), green wastes and a screened compost (green waste/kitchen waste compost), using the COMPOCHIP microarray. The composting process showed a typical temperature development, and the highest degradation rates occurred during the first 14 days of composting, as seen from the elevated CO_2_ content in the exhaust air. With an exception of elevated nitrite and nitrate levels in the day 34 samples, physical–chemical parameters for all compost samples collected during the 63 day process indicated typical composting conditions. The microbial communities changed over the 63 days of composting. According to principal component analysis of the COMPOCHIP microarray results, compost samples from the start of the experiment were found to cluster most closely with the digestate and screened compost samples. The green waste samples were found to group separately. All starting materials investigated were found to yield fewer and lower signals when compared to the samples collected during the composting experiment.

## Highlights

• Highest degradation rates in compost in first 14 days.
• Similar microbial communities in digestate and screened compost samples.
• Fewer and lower signals in all starting materials than in day 20, 34 and 63 composts.
• Compost microbial communities changed over 63 day composting process.

## 1 Introduction

In order to reduce negative impacts on the environment, the European Landfill Directive (1999/31/EC) states that by 2016, the disposal of biodegradable municipal waste should be reduced by 75%, compared to 1995 values. Composting of municipal, agricultural and industrial wastes is among the most commonly used biowaste treatment options employed across Europe. Another increasingly used technology is anaerobic digestion (AD), whereby organic substrates are converted into a methane rich biogas, suitable for heat and electricity production. A digestate remains at the end of the process, which contains both undegraded and non-degradable organic compounds as well as nutrients (Körner et al., 2010). Recently, the combination of both anaerobic digestion and composting for biowaste treatment has been increasingly promoted. The advantage is the combined generation of energy and material products – biogas and compost as a soil improver. This combination increases the efficiency of bioresource utilisation. However, before integrating an anaerobic digestion unit into an existing composting facility, the economic framework and technical setup has to be evaluated and optimised.

Process optimisation is important for both anaerobic digestion and composting facilities, as well as for plants integrating both processes. Digestates are often characterised by a high biogas potential, indicating an inefficient anaerobic digestion process. For instance, Linke et al. (2007) reported a remaining biogas potential in digestates from a dry fermentation plant using maize silage and turkey manure from approximately 25 NL biogas per kg digestate fresh matter. For comparison, the actual biogas production during anaerobic digestion was around 100 NL biogas per kg fresh input. Balsari et al. (2010) investigated the methane yields from the mechanically separated solid fractions of digestates from 6 biogas plants and found variations from 50 L methane production per kg volatile solids of up to around 210 L. They suggested reuse in the biogas plant to increase the overall process efficiency.

The remaining, undegraded organic products can also be subjected to composting, although composting could as well be conducted with more efficiently treated digestates. The composting of digestates differs from the composting of common substrates, since the digestates are often characterised by very low dry matter content (dry matter content of 20–26% for digestates investigated by Linke et al. (2007). In a study conducted by Bustamante et al. (2013), the composting of pig slurry digestate with different bulking agents was investigated, and stable and mature composts were obtained. A similar study by Bustamante et al. (2012) used the solid fraction of a digestate from the anaerobic co-digestion of cattle slurry and silage, with or without vine shoot prunings as a bulking agent, in a composting experiment. The composts obtained showed adequate degrees of stability and maturity, suitable physical properties for use as growing media, and were capable of the suppression of the plant pathogen *Fusarium oxysporum* f. sp. *melonis*.

Aerobic conditions are needed for composting processes (Körner, 2008), and the addition of aerobic microorganisms from co-substrates can help in the composting process. Mixing composts with drier and more bulky materials is necessary to provide suitable composting conditions. Since microorganisms play a major role in anaerobic digestion as well as composting, knowledge on the behaviour and dynamics of microbial communities is necessary for any kind of process optimisation (Sundberg et al., 2011). This is because the presence of different bacteria can positively or negatively affect the composting process, and modification of the type and amount of input materials can change the microbial communities, and the composting process. In recent years, the microbiology of composting processes has been heavily investigated, both with classical (Kausar et al., 2011; Lv and Yu, 2013), physiological (Mondini and Insam, 2005) and molecular (Tiquia et al., 2005; Franke-Whittle et al., 2009; Yamamoto et al., 2011) approaches. However, knowledge regarding the microbial communities involved in anaerobic digestion is still limited, and that of combined processes is even more limited.

A microarray targeting plant, animal and human pathogens, plant disease suppressive bacteria, as well as microorganisms that have been previously reported in the composting process, was developed by Franke-Whittle et al. (2005, 2009). The COMPOCHIP microarray allows the quick detection of many different microorganisms in a single test, and has been used in several composting studies (Danon et al., 2008; Cayuela et al., 2009; Sundberg et al., 2011, 2013; Fritz et al., 2012).

The aim of this study was to investigate the changes in microbial communities in a composting experiment using the COMPOCHIP microarray. Three input substrates were selected: a municipal food waste digestate, a green waste and a screened compost produced from green waste and kitchen waste. Of interest was to determine how the microbial composition would evolve during the composting process.

## 2 Materials and methods

### 2.1 Substrates for composting

Fresh green waste (*Gw*), screened compost (*Co*) and digestate (*Dig*) were used as input substrates for the experiment (Table 1). *Gw* was produced from wood chips, yard trimmings and tree cuttings and taken from a composting facility. It was shredded to a size appropriate for laboratory reactor trials. *Gw* was expected to contain ubiquitous microrganisms. *Co* was produced from separately collected organic waste (green waste and kitchen waste) by means of an enclosed reactor technology, following the guidelines of the German biowaste ordinance (BioAbfV, 1998). After composting, it was screened and the 20–50 mm fraction was used in this study. *Co* was used in order to introduce a variety of aerobic microorganisms into the mixture, which are typical for composting. *Dig* was produced by an industrial mesophilic anaerobic digestion process. The digester was fed with the liquid fraction derived from shredded food waste separated by means of a mash-separator, as well as with oil residues from the olive oil industry. The mixing ratio (food waste liquid: olive residues) was 9:1 (fresh weight). *Dig* was expected to contain a predominantly anaerobic microbial flora.

A mixture of the three substrates was manually prepared for the composting experiment. One of the purposes of the mixing was to introduce a significant share of microorganisms from all fractions into the composting substrate. The water content of the *ISM* (initial starting material) was adjusted to 64% by the addition of the anaerobic digestate, as seen in Table 1.

### 2.2 Composting and sampling

**Fig. 1:**
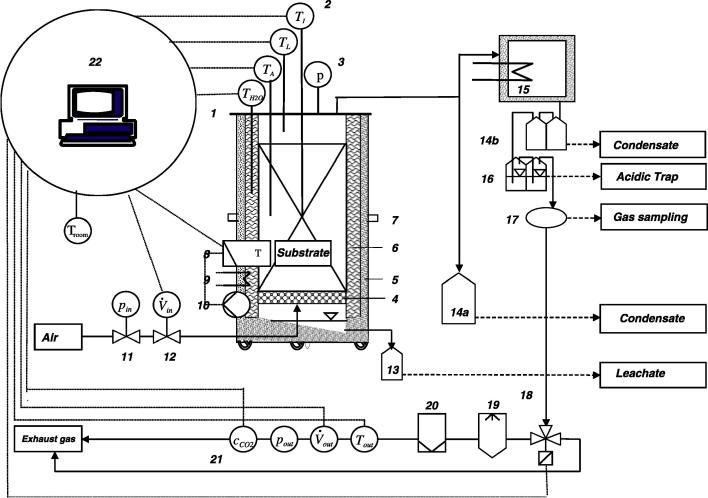
Schematic set up of the composting unit. *Note:* 1 – reactor lid, 2 – temperature sensors, 3 – manometer, 4 – sieve lid, 5 – insulation, 6 – water jacket, 7 – tipping device, 8 – control box, 9 – heating system, 10 – water pump, 11 – pressure reducer, 12 – mass flow meter with regulating valve, 13 – leachate collection, 14a, b – condensate collection, 15 – refrigerator, 16 – acidic traps, 17 – gas sampling unit, 18 – switching valve, 19 – liquid separator, 20 – air filter, 21 – measurement units for temperature, gas mass flow, pressure, carbon dioxide, 22 – electrotechnical unit for process regulation and data collection, *c*_CO2_-, *p_out_*-, *V_out_*-, *T_out_*.

Composting was carried out in an insulated 100-L steel tank composting unit, which is described in detail in Körner (2008). The schematic set-up of the whole unit, including peripheral equipment is presented in Fig. 1. In total, three composting experiments were carried out, each in duplicate. All experiments showed the typical course of composting. Samples from one of the experiments was chosen for investigation of the microbial consortia.

The composting reactor was filled with 55.6 kg fresh matter (fm) of the substrate mixture and was aerated at a rate of 100 L h^−1^. For that purpose, compressed air was bubbled from underneath the mixture to oxygenate the substrate. The gas flows were manually adjusted and continuously monitored during composting by means of a mass flow meter. The composting period lasted 63 days and during this period, the substrate mixture was turned three times (after 8, 20 and 34 days). Turnings were performed by emptying the reactor and manually mixing the material. Three representative samples were taken after mixing and reactors were refilled. The samples were either analysed directly, or stored (4 °C and −20 °C) for future analyses.

The weight losses along the composting process were determined by weighing the whole reactor on each turning day. Furthermore, the amounts of leachate were measured upon turning. The temperature profiles of the substrate mixtures during composting and of the gaseous phase above the substrate were monitored several times a day with PT 100 temperature probes. No additional heat was provided. The exhaust air was captured at the top of the reactor and sent to a waste gas treatment system. The gas treatment system consisted of a condenser and an acid trap, and the condensate and acidic solution were analysed on demand regarding NH_3_-content. An air stream was firstly conducted through bottles placed in a refrigerator to remove NH_3_-containing condensed water. Air was then led through 2 bottles with gas dispensers which contained 0.5 N H_2_SO_4_ as a scrubbing solution and a color indicator to show when the solution became saturated. After saturation, the scrubbing solution was exchanged. The composition of the remaining gaseous emissions (CO_2_, O_2_, CH_4_, N_2_, H_2_, N_2_O) was measured every 2–3 days by gas chromatography.

Samples were collected for analysis at the start of the experiment (*ISM*), after 8 days, after 20 days (fresh compost; *FC*), after 34 days (slightly matured compost; *SMC*) and after 63 days (matured compost; *MC*). Furthermore, the three substrates (*Gw*, *Dig*, and *Co*) were analysed prior to mixing.

### 2.3 Physical–chemical parameters

The dry matter content (dm) of triplicate samples was assessed according to DIN EN 12880 (2001). The drying step was performed at 105 °C for at least 24 h and dried samples were ground to a particle size <0.25 mm. Total Kjehldahl nitrogen (TKN) of samples was determined using a Kjeldahl catalyst at 380 °C to mineralise organic N to ammonia, followed by a distillation step under a vapor stream, basification with NaOH and N titration in a H_3_BO_3_ solution. Total organic carbon (TOC) was measured by the dry combustion method at 550 °C.

Ammonia/ammonium, nitrate, nitrite concentration and pH were determined in eluates prepared using demineralised water (DIN 38414-4, 1984). pH was measured according to DIN 38404-5 (2005), ammonia/ammonium concentration according to DIN EN ISO 11732 (2005), nitrate and nitrite concentration according to DIN EN ISO 10304-1 (2009), and total soluble nitrogen according to DIN 38409-H 28 (1992).

The ammonia/ammonium concentrations in the condensed water and acidic trap solution were determined using a gas sensitive electrode as described for the eluates. The gas composition in the exhaust gas was measured by gas chromatography. The methods are explained in more detail in Körner (2008).

### 2.4 Molecular methods for determination of microorganisms

The starting substrates *Gw*, *Dig*, *Co* and the samples from the composting processes *ISM*, *FC*, *SMC* and *MC* were analysed regarding their microbial composition using molecular methods. The COMPOCHIP microarray, spotted with 414 probes targeting compost-relevant microorganisms, including plant, animal and human pathogens, and bacteria related to plant disease suppression, was used. The PowerSoil DNA Isolation Kit (MO BIO Laboratories, Carlsbad, California, USA) was used to extract DNA from the different composts and starting materials. For a few target microorganisms, only one or two probes were printed on the array, but for most target microorganisms, at least three probes were printed on the array.

The specificity of all probes was assessed using the ARB program (Ludwig et al., 2004). The majority of probes printed on the microarray were tested using pure cultures of microorganisms, and shown to work well with only a low percentage of non-specific hybridisations (Franke-Whittle et al., 2005, 2009). All the probes included on the COMPOCHIP microarray were designed to have similar melting temperatures, and probe sequences ranged in length from 17 to 25 nucleotides.

DNA extraction from substrate and compost samples, fluorescence labelling of target DNA, hybridisation, scanning of arrays were conducted as described by Franke-Whittle et al. (2005, 2009). Principal component analysis (PCA) of the total signal-to-noise ratio (SNR) from microarray data of the study was conducted using CANOCO for windows 4.5 (Ter Braak and Smilauer, 2002). The signal-to-noise ratio (SNR) for all spots was calculated according to Loy et al. (2002). Signals were assumed to be positive if an SNR value of ⩾2 was obtained (Loy et al., 2002).

## 3 Results and Discussion

### 3.1 Composting process

#### 3.1.1 Initial substrate mixture (ISM)

Each of the three starting materials (*Gw*, *Co*, *Dig*) used in the composting process served different functions, and thorough mixing of the materials was essential due to their non-homogenous natures (especially *Gw* and *Co*). Liquid digestate was used to adjust the water content to 64% in the *ISM*, slightly higher than the optimal range for composting (50–60% moisture content; Tiquia et al., 1996; Gajalakshmi and Abbasi, 2008). The digestate also provided the composting experiment with an anaerobic community that had developed during the earlier anaerobic process. The *Gw* material gave stability and structure to the mixture, and allowed good air permeability. It is also expected that the *Gw* material will have provided an additional microbial community, from the surroundings, due to the large surface area of plant materials. The screened compost, *Co*, provided an aerobic microbial community which had developed during composting.

#### 3.1.2 Composting process

**Fig. 2:**
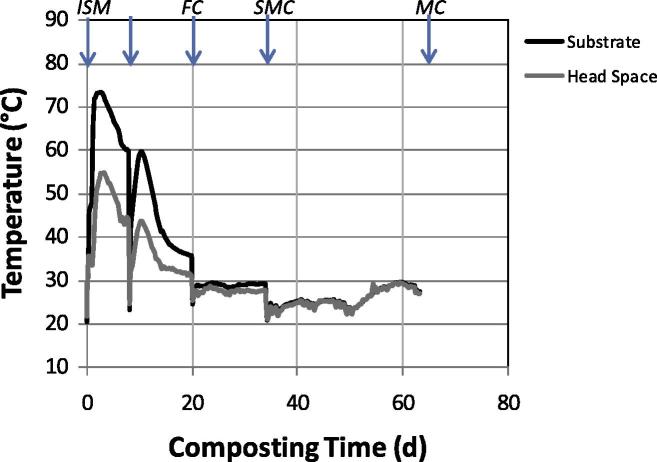
Temperature profile of composting process und samples taken during the process for microbiological analyses: *ISM* (initial substrate mixture), *FC* (fresh compost), *SMC* (slightly mature compost), *MC* (mature compost). *Note:* The arrows indicate turning and sampling days.

Basic data describing the composting process are presented in Table 2. After 1 week of composting, almost 2 L of leachate (3.6% of the input mass) had been produced (Table 2). During composting, a 19% weight loss of the wet mass occurred. This reduction can mainly be attributed to the transformation of organic matter into carbon dioxide and water vapour, but also, to a lesser extent, to the leaching of liquids from the substrate. Leachate formation, as well as the water content of the compost, showed that the moisture content was at the upper level suitable for composting during the whole composting period. The highest degradation rates occurred in the first 2 weeks of composting, as can be seen from the CO_2_ content in the exhaust air. This indicates a rapid start of the composting process. After three weeks it declined to below 1% (v/v), indicating that the major degradation was over. Water evaporation was also high in the first phases, due to the elevated temperatures (up to 73 °C; Fig. 2) and contributed to the loss of wet mass.

Composting microorganisms consume O_2_. In this experiment, O_2_ was provided by aeration at a rate of 100 L air per hour, and strong aerobic activity occurred within the first two weeks. This could be recognized by the drop in O_2_ concentration in exhaust gas from 21% (v/v), which was the concentration in the inlet air, to 11% after one week. By the end of the second week, aerobic activity slowed down and O_2_ concentration in exhaust gas increased to 17%. O_2_ concentrations of 19% and over in the exhaust gas were detected after two weeks.

Although composting occurs aerobically, anaerobic niches can form. This may lead to the production of CH_4_ by methanogenic microorganisms introduced into the compost from the anaerobic digestates. Traces of CH_4_ (up to 0.08%, v/v) were detected in the first two weeks of the composting process, indicating a very low activity of methanogenic organisms during this period. Cuhls et al. (2008) also detected CH_4_ emissions during composting, depending on the process conditions and substrate type.

N_2_O formation may occur during composting. Körner (2008) reported that N_2_O can sometimes be formed during the later stages of the composting process. Formation of N_2_O is an indication of incomplete denitrification and low O_2_ concentrations can induce it. N_2_O production during conventional composting was also reported by Hellmann et al. (1997), Sommer et al. (1999) and He et al. (2001). During the experiments of this study, traces of N_2_O (<0.03%, v/v) were detected in weeks 5 and 6, indicating incomplete denitrification activities, which probably occurred in anaerobic niches in the compost.

The temperature graph of the composting trial (Fig. 2) shows a typical development for composting processes containing the phases of self-heating, cooling and stabilisation (Insam et al., 2010). The sudden temperature drops in the graph after one, three and five weeks can be attributed to heat removal from the compost due to the turning activity. Composting started with a thermophilic phase, in which temperatures higher than 70 °C were obtained for two days. The short self-heating phase (six days above 65 °C) indicates that low amounts of easily degradable substances were present in the *ISM* and that they were quickly consumed by microorganisms. Sometimes short self-heating phases may occur, when working with small scale systems due to heat losses to the surroundings. This was not the case in our study, since the 100 L reactors utilised were well insulated and contained a water filled double wall. The wall temperature represented the temperature in the middle of the substrate mixture and automatic heating took place when heat losses occurred. The set-up simulated self-heating of larger systems. The reason for the short self-heating phase has rather to be found in the substrate, as shown also by the comparison with the temperature profiles in experiments composting different mixtures of substrates (Bustamante et al., 2012). After the easily degradable substances were consumed, temperature decreased slowly to air temperature over a period of approximately two weeks. The recommendation for hygienisation of composts according to BioAbfV (1998) state that temperatures >65 °C should be sustained for at least seven days or >55 °C for 14 days. Taking into account that this guideline refers to commercial composting facilities treating non-homogenous materials, we can consider that the compost produced in this lab trial was hygienically acceptable.

No significant degradation was seen in the compost during the stabilisation phase (day 20 until the end of composting), but presumably, organic compound rearrangement reactions (humification) occurred. The stabilisation phase commonly takes place at ambient temperatures (Insam et al., 2010). A slight temperature increase occurred in the experiment at the end of the stabilisation phase, indicative of a microbiologically active community. This increase can probably be attributed to the development of a fungal flora which was able to metabolise the more recalcitrant components. The metabolites arising from these breakdown processes can in turn also be metabolised by bacteria, and this metabolism probably resulted in the temperature increase that can be seen in Fig. 2.

Table 3 shows selected physical–chemical parameters of the different samples from the composting process. The dry matter content decreased during the thermophilic phase and again increased with the cooling of the substrate. These values are relative, and not absolute values. The dry matter decrease is attributed to relative water enrichment due to substrate degradation and generation of process water formation. The dry matter increases are attributed to water losses due to aeration. The water content was at the upper limit for optimal aerobic processes. This indicates that there were enough niches present for both aerobic as well as anaerobic microorganisms.

The highest pH values were found during the thermophilic and early cooling phases. Nonetheless, pH values were in a suitable range (between 7 and 8.5) for the optimal growth of many microorganisms for the duration of the composting process. Included are ammonifiers (5.0–8.0), autotrophic nitrifiers (6.5–8.0) and denitrifiers (6.0–8.0) (Knowles, 1982; Scheffer and Schachtschabel, 2002; Körner, 2008).

Water soluble nitrogen comprises ammonia/ammonium, nitrate and nitrite (Table 3). Nitrate and nitrite were not detected in any of the starting substrates or any compost, with the exception of the compost collected on day 34. ${\text{NO}}_{3}^{-}$ is produced by the nitrification of ammonia, while ${\text{NO}}_{2}^{-}$ is an intermediate product in this process. Autotrophic and heterotrophic nitrifying microorganisms are known to be active in composting processes (Körner, 2008). The lack of ${\text{NO}}_{3}^{-}$ or ${\text{NO}}_{2}^{-}$ does not necessarily mean that nitrifiers were inactive; more likely, ${\text{NO}}_{3}^{-}$ was being simultaneously denitrified into N_2_. The ${\text{NO}}_{3}^{-}$ accumulation at day 34 was likely a result of the very narrow milieu conditions regarding temperature, O_2_ content and organic N concentration in the compost. Temperatures between 25 and 35 °C, O_2_ >19%, and organic N >2% of compost dm, as seen at day 34, provide optimal conditions for ${\text{NO}}_{3}^{-}$ accumulation in compost (Körner, 2008). Prior to day 34, temperatures were too high.

Ammonia/ammonium is formed from the ammonification of proteins, and the process increases also the soluble organic nitrogen in compost. In common composting processes, maximum ammonia/ammonium levels occur during the thermophilic phase and generally range between 0.09% and 1.8% of the dm. The level predominantly depends on the organic N content of the initial substrate (Körner, 2008). In this experiment, the ammonia/ammonium levels were already relatively high at the start of the composting process as a result of the digestate which was rich in ammonia/ammonium (Table 1). The maximum ammonia/ammonium peak was 0.36% of the dm at day 20, while at the end of the composting process, the value was 0.03% of the dm. According to Körner (2008), typical final ammonia/ammonium values are between 0.02% and 1.0% of the dm, indicating normal ammonia amounts in the mature compost in this study.

The slight increase in the total organic nitrogen content (TKN) in the compost is a result of N-losses via exhaust air and leachate on the one hand, and N enrichment due to carbon degradation on the other. In this experiment, the N peak was at day 63, with 2.1% of dm.

The total organic carbon (TOC) in the material decreased clearly within the first week, and showed no drastic changes in the remaining period (Table 3). This indicates that degradation processes were most significant in the first week. The changes in mass related to initial wet matter are given in Table 2. The highest changes also occurred during the first week. Beside organic matter degradation, a further influencing factor was the formation of leachate. To evaluate organic matter degradation, the degradation rate of the initial dry matter content was calculated at 28% in the first week, and increased up to 37% by the end of the composting period.

### 3.2 Microarray analysis

The COMPOCHIP 16S rRNA gene microarray was applied to directly determine which microorganisms were present in the different compost samples. The microarray contains a limited set of probes (414) specific to microorganisms known to be pathogens, as well as microorganisms considered to be important in composting processes. In total, 96 different microorganisms were detected in the 7 samples (Table 4). Because a linear correlation between target concentrations and signal intensities of the various probes has been reported by others (Taroncher-Oldenburg et al., 2003; Tiquia et al., 2004), the results can be considered semi-quantitative. The signal-to-noise ratios (SNRs) obtained after hybridisation of the COMPOCHIP microarray were determined, and are presented in Table 4.

**Fig. 3:**
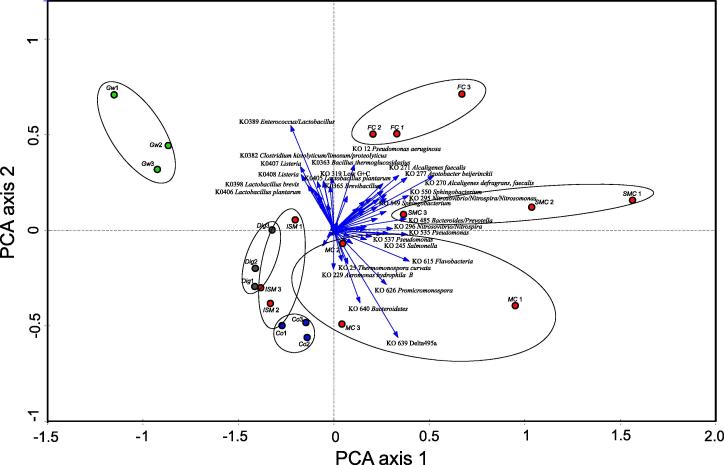
Principal component analysis (PCA) loading plot obtained by principal component analysis, depicting the organisms responsible for community differences amongst the samples. The lengths of the vectors indicate their significance for compost differentiation (longer arrow length means higher SNR of probe).

Fig. 3 shows an ordination graph, where the two relevant axes explain 56.9% of the variance. The plot indicates the organisms (probes) which were responsible for community differences amongst the composts and starting materials (indicated by arrows). Multivariate analysis revealed that the bacterial community compositions of the various starting materials differed, and that the composts made from them changed over time. Replicate samples were found to mostly group closely together.

The probes KO 389 (*Enterococcus*/*Lactobacillus*), KO 639 (Delta proteobacteria), KO 277 (*Azotobacter beijerinckii*), KO 270 and KO 271 (*Alcaligenes defragrans*, *faecalis*) and KO 295 *Nitrosovibrio*/*Nitrospira*/*Nitrosomonas* had the highest discriminatory capacity of all probes, and allowed the differentiation of samples.

The freshly prepared *ISM* samples (day 0) were found to cluster most closely with the digestate and screened compost samples, indicating similar microbial communities. Interestingly, the microbial communities of the green waste samples were found to be more different, despite the *ISM* samples being comprised of 52.4% (fm) green waste. One possible explanation for this finding is that a majority of the organisms found in green waste did not have probes represented on the array.

Fewer and lower signals were detected in the starting materials (*Co*, *Dig*, *Gw*) and *ISM* samples (day 0) compost than in the other compost samples (*FC*, *SMC*, *MC*) collected during the experiment. This would indicate lower bacterial numbers and diversity; however, it is also possible that probes specific for many of the microorganisms present in these samples were not present on the microarray. Another explanation is that these starting materials contain diverse communities of microorganisms of which the majority are present in numbers below the detection limit of the microarray. As green waste composts are higher in recalcitrant lignocellulosic materials, it would be expected that diverse communities of degrading bacteria and fungi should be present.

The microbial communities of the composts were seen to change over the 63 days of composting. Interestingly, there were more and higher signals upon hybridisation of the array with DNA extracted from the composts after 20 and 34 days of composting (*FC*, *SMC*) than in all other composts and input materials investigated. Again, it is possible that these mesophilic organisms are represented on the COMPOCHIP to a higher degree than microorganisms found in the very early and late stages of the composting process.

Of concern was the finding of *Salmonella* in the *FC* and *SMC* composts. The presence of these microorganisms in an end product would indicate an inefficient sanitation step, as animal, human and plant pathogens should normally be eliminated through high temperatures and through microbial succession in the composting process (Sundberg et al., 2013). The short thermophilic hygienisation phase which occurred in the composting process is most likely the reason for the survival of this pathogen. However, signals below the detection limit for the *Salmonella* probe were found in the two of the three *MC* compost samples. This indicates that besides temperature, other factors may be important for hygienisation. One such factor may be the production of antibiotics by actinobacteria. *Thermomonospora*, a genus in the phylum Actinobacteria, was indeed detected in the mature compost samples. On the other hand, *Listeria*, a genus which also contains human pathogens, was present in the green waste samples, but was quickly reduced during the process and completely eliminated from the final compost products. It would seem that *Listeria* is more heat sensitive than *Salmonella.*

Various *Clostridium* probes (*Cl. fallax*/*perfringens*-KO 264, *Cl. formicoaceticum*-KO 380, KO 381 and *Cl. histolyticum*/*limosum*/*proteolyticum* KO 382) gave positive hybridisation signals in the starting materials. *Clostridium* is a genus of obligate anaerobic, spore forming organisms, and their presence in soil and composts in not unusual. During composting, the conditions were in general aerobic due to the aeration applied. However anaerobic zones may have formed due to inhomogeneous air flow, evidenced by the detection of methane during the first two weeks, and the detection of N_2_O in weeks 5 and 6. The genus *Clostridium* contains different pathogens. However, with the exception of the *Cl. fallax*/*perfringens* probe, no signals above the detection limit were detected in the compost after 63 days, thus alleviating potential concerns. *Cl. botulinum* was not detected in any sample. This organism has been reported to occur occasionally in composts and can cause serious illness, including death (Böhnel et al., 2002).

Different *Lactobacillus* probes were found to yield signals in the green waste (*Lactobacillus*-KO 320, *L. brevis*-KO 398 and *L. plantarum*-KO 405, KO 406) and in the digestate (*L. panis*-KO 506). *Lactobacillus* species are typically found in the very early phases of composting (Alfreider et al., 2002; Partanen et al., 2010) and produce large amounts of lactic acid. Lactic acid and other acid producing bacteria have also been reported in anaerobic digestion processes (Shin et al., 2010; Probst et al., 2013; Sundberg et al., 2013).

The probes targeting the autotrophic nitrification bacteria *Nitrospira*, *Nitrosovibrio* and *Nitrosomonas* were found to hybridise with DNA from the *FC*, *SMC* and *MC* composts, as well as with the compost starting material (*Co*). These bacteria have also been reported in composts from other studies (Kowalchuk et al., 1999; Danon et al., 2008; Cayuela et al., 2009; Bougnom et al., 2012). They are known to be active in the first step of autotrophic nitrification in which ${\text{NH}}_{4}^{+}$ becomes oxidised to ${\text{NO}}_{2}^{-}$. *Nitrospira* is also active in the second step of autotrophic nitrification, where ${\text{NO}}_{2}^{-}$ is oxidatively converted into ${\text{NO}}_{3}^{-}$ (Rheinheimer et al., 1988; Körner, 2008). As long as ${\text{NH}}_{4}^{+}$ and O_2_ are contained in the substrate, autotrophic nitrifiers should be active. The accumulation of ${\text{NO}}_{3}^{-}$ in the day 34 compost is most likely the result of the milieu conditions limiting denitrification of ${\text{NO}}_{3}^{-}$ into N_2_ (Körner, 2008).

Nitrification can also occur in heterotrophic microorganisms. Fungi such as *Aspergillus* and *Penicillium*, and species of the bacteria *Pseudomonas*, *Bacillu*s and *Streptomyces* are known heterotrophic nitrifiers (Verstraete, 1975; Killham, 1986; Körner, 2008). *Bacillus* species occurred in *Gw* only and lower SNR values for this bacterium were obtained in the composts as the process continued. *Streptomyces* was only detected (low SNR) in the *MC* sample. *Pseudomonas* species were not found in *Gw*, but in *Dig* and *Co*. They increased during the composting process and yielded the highest SNR values in the *SMC* sample. This sample was also the only one which also contained ${\text{NO}}_{3}^{-}$ (Table 3). *Pseudomonas* species are ubiquitous in the environment and have been previously reported in mature compost bacterial communities (Ryckeboer et al., 2003a; Danon et al., 2008). *Pseudomonas* strains are capable of N_2_ fixation, denitrification and the degradation of pollutants (Lalucat et al., 2006), and several strains are known to confer plant-disease suppressiveness (Haas and Défago, 2005).

The genera *Azotobacter* and *Clostridium* include members that are capable of asymbiotic nitrogen fixation (Müller, 1965; Schlee and Kleber, 1991; Körner, 2008). Nitrogen fixing bacteria were found in composting samples by de Bertoldi et al. (1983), Safwat (1980) and Diaz-Ravina (1989). Probes targeting both *Azotobacter* (KO 276, KO 277, KO 278) and *Clostridium* (KO 264, 380, 381, 382, 383, 225) were found to hybridise with DNA from various samples in this study. *Azotobacter* spp. was not found in the initial substrates, but developed during composting and was found in the *FC*, *SMC* and *MC* compost samples. The highest signals for *Azotobacter* were found on day 34 in the *SMC* sample. *Azotobacter*, a genus of soil microbes able to fix atmospheric nitrogen, was also found in different compost types and composts of different ages in a study conducted by Cayuela et al. (2009). Although nitrogen fixing bacteria are often found in composting and were also detected in our study, their presence does not necessarily mean that nitrogen fixation is occurring. Calculations from Körner (2008) indicate that nitrogen fixation cannot occur at a significant rate during composting processes, due to a surplus of N-compounds in the substrate and a limitation of available carbon. Loveless (1999) suggests that values of 180 mg L^−1^ N in solution result in the inhibition of nitrogenases. In our experiments, the total soluble N was between 199 and 657 mg L^−1^ during the entire composting period. This indicates that the nif genes of bacteria detected in this study were most likely not being expressed.

Positive hybridisation signals were obtained for the *FC*, *SMC*, *MC* composts with the probes targeting *A. faecalis*/*defragrans* (KO 270, 271, 350). This is despite this organism not being detected in any of the raw compost materials. Possibly, it was present in numbers below the detection limit in one of the raw compost materials, and under the conditions of composting was able to increase in numbers so to be detected by the microarray. Despite its name, *A. faecalis* is not typical to faeces, but is a common non-pathogenic, environmental bacterium which has been reported to be present in composts by other authors (Droffner et al., 1995; Ryckeboer et al., 2003a; Danon et al., 2008).

The genus *Bacillus* has been previously reported to be the most abundant group of bacteria in compost during the thermophilic phase and throughout the entire composting process (Ryckeboer et al., 2003b). The genus contains free-living and pathogenic species, which under stressful environmental conditions, such as high temperature, can produce endospores that can stay dormant for extended periods (Madigan and Martinko, 2005). Interestingly, in this study, low levels of *Bacillus* were found in the green waste, but not in any other of the composts investigated, including compost sampled in the thermophilic phase. It would seem that these species of *Bacillus* were outcompeted by the other bacteria present in the composting process.

Certain strains of *Xylella fastidiosa* are individually mentioned in the EU Directive 77/93. The organism is known to cause disease to grapevine. However, its occurrence has only been reported in Asia and America, and thus far, not in Europe. *X. fastidiosa* is only likely to establish and grow in warmer regions, and in Europe, grape-growing areas including the southern Iberian and Italian peninsulas and the lowlands of Greece (EPPO, 2013). Since the green waste and the compost came from Germany, and the digestate was from food waste in a wine-growing region of Italy, we can assume that *X. fastidiosa* originated from the *Dig* samples*.* It was, however, not found in the native compost.

## 4 Conclusions

This study investigated the physical–chemical parameters and the microbial communities involved in a composting process of a mixture of anaerobic digestate, green waste and screened compost. Degradation rates in the compost were highest in the first 14 days of the process, and the composting process showed a typical temperature development. Standard composting conditions were evident according to the physical–chemical parameters measured from all composts. Microarray analysis indicated changes in the compost microbial communities over the 63 day composting process, and fewer and lower signals were obtained for all three starting materials than in the composts collected after 20, 44 and 63 days. Similar microbial communities were found in the digestate and screened compost samples, while the microbial community of the green waste clustered distinctly. Microorganisms detected in the green waste but not in other samples and composts included species of *Bacillus*, *Clostridium*, *Lactobacillus* and *Listeria.* The mature composts grouped more closely with the initial substrate mixtures, than with the fresh and semi-mature composts. Probes specific for *Enterococcus*/*Lactobacillus*, *Delta proteobacteria*, *Azotobacter beijerinckii*, *Alcaligenes defragrans*, *faecalis* and *Nitrosovibrio*/*Nitrospira*/*Nitrosomonas* had the highest discriminatory capacity of all probes, and allowed the differentiation of samples. This study has shown the feasibility of using a combination of anaerobic digestion and composting for the treatment of organic waste products. However, it is important that the period of self-heating is long enough to ensure sufficient hygienisation of the input materials used.

## Figures and Tables

**Table 1:** Physical and chemical parameters of original substrates and of the mixture used in the composting experiment.

Sample	Amount (% fm)	Amount (% dm)	Dry matter (% fm)	pH	${\text{NH}}_{4}^{+}$ (mg L^−1^)	Total water soluble N (mg L^−1^)	TKN (% dm)	TOC (% dm)
Green waste (*Gw*)	52.40	64.40	48	5.54	69	235	1.94	37.05
Compost (*Co*)	21.50	33.30	60	7.74	74	309	1.55	25.75
Digestate (*Dig*)	26.20	2.30	6	7.79	513	654	1.40	39.65
Initial substrate mixture (*ISM*)	–	–	36	7.10	120	266	1.87	34.63

*Note:* fm – fresh matter; dm – dry matter; TKN – Total Kjeldahl nitrogen; TOC – Total organic carbon; ${\text{NO}}_{3}^{-}$ and ${\text{NO}}_{2}^{-}$ – under detection limit (0.05 mg L^−1^ eluate); ${\text{NH}}_{4}^{+}$ and Total water soluble N refer to the content in the eluate (both are water soluble fractions).

**Table 2:** Parameters describing the composting process.

Compost	Composting age (days)	Wet mass after turning (kg)	Cumulative sampling losses (kg)	Leachate (mL)	Wet mass losses^a^ (% of initial mass)	Water content (% fm)	CO_2_ content in headspace (vol%)
Initial substrate mixture (*ISM*)	0	55.6	0.0	0	0.0	63.9	0.03
	8	49.5	1.8	1980	7.6	71.8	9.95
Fresh compost (*FC*)	20	45.1	4.1	480	11.4	69.2	1.51
Slightly matured compost (*SMC*)	34	43.1	4.9	52	13.7	66.9	1.00
Mature compost (*MC*)	63	40.0	4.9	165	19.2	71.8	0.45

fm – Fresh matter.

a The sampling losses were considered.

**Table 3:** Physical–chemical parameters of samples taken during composting.

Compost	Composting age (days)	Dry matter (% fm)	pH	${\text{NH}}_{4}^{+}$ (mg L^−1^)	Total soluble N (mg L^−1^)	${\text{NO}}_{2}^{-}$ (mg L^−1^)	${\text{NO}}_{3}^{-}$ (mg L^−1^)	TKN (% dm)	TOC (% dm)
Initial substrate mixture (*ISM*)^a^	0	36	7.1	120	266	nd	nd	1.9	34.6
	8	29	8.5	118	287	nd	nd	1.9	26.0
Fresh compost (*FC*)^a^	20	31	9.0	233	657	nd	nd	nm	26.7
Slightly matured compost (*SMC*)^a^	34	33	7.8	82	283	80	500	nm	nm
Mature compost (*MC*)^a^	63	34	7.5	17	199	nd	nd	2.1	28.7

*Note:* fm – fresh matter; dm – dry matter; nd – not detected; nm-not measured. ${\text{NH}}_{4}^{+}$, ${\text{NO}}_{2}^{-}$, ${\text{NO}}_{3}^{-}$ and total soluble N refer to the content in the eluate; all are water soluble fractions.

a Samples from composting process used in COMPOCHIP analyses.

**Table 4:** Microarray results showing SNR values of the different starting materials and compost samples. The green represents hybridisation signals with SNR values above the threshold of 2.
